# Silencing of *Sporothrix schenckii GP70* Reveals Its Contribution to Fungal Adhesion, Virulence, and the Host–Fungus Interaction

**DOI:** 10.3390/jof10050302

**Published:** 2024-04-23

**Authors:** Luz A. López-Ramírez, José A. Martínez-Álvarez, Iván Martínez-Duncker, Nancy E. Lozoya-Pérez, Héctor M. Mora-Montes

**Affiliations:** 1 Departamento de Biología, División de Ciencias Naturales y Exactas, Campus Guanajuato, Universidad de Guanajuato, Guanajuato Gto. 36050, Mexico; adrianalr@ugto.mx (L.A.L.-R.); martinezjose@ugto.mx (J.A.M.-Á.); nelppat@hotmail.com (N.E.L.-P.); 2 Laboratorio de Glicobiología Humana y Diagnóstico Molecular, Centro de Investigación en Dinámica Celular, Instituto de Investigación en Ciencias Básicas y Aplicadas, Universidad Autónoma del Estado de Morelos, Cuernavaca Mor. 62209, Mexico; duncker@uaem.mx

**Keywords:** fungal cell wall, cytokine production, adhesins, granulocytes, phagocytosis, *N*-linked glycans, virulence factor, β-1,3-glucan, innate immune sensing

## Abstract

*Sporothrix schenckii* is one of the etiological agents of sporotrichosis, a cutaneous and subcutaneous infection distributed worldwide. Like other medically relevant fungi, its cell wall is a molecular scaffold to display virulence factors, such as protective pigments, hydrolytic enzymes, and adhesins. Cell wall proteins with adhesive properties have been previously reported, but only a handful of them have been identified and characterized. One of them is Gp70, an abundant cell wall protein mainly found on the surface of yeast-like cells. Since the protein also has a role in the activity of 3-carboxy-*cis*,*cis*-muconate cyclase and its abundance is low in highly virulent strains, its role in the *Sporothrix*–host interaction remains unclear. Here, a set of *GP70*-silenced strains was generated, and the molecular and phenotypical characterization was performed. The results showed that mutants with high silencing levels showed a significant reduction in the adhesion to laminin and fibrinogen, enzyme activity, and defects in the cell wall composition, which included reduced mannose, rhamnose, and protein content, accompanied by an increment in β-1,3-glucans levels. The cell wall *N*-linked glycan content was significantly reduced. These strains induced poor TNFα and IL-6 levels when interacting with human peripheral blood mononuclear cells in a dectin-1-, TLR2-, and TLR4-dependent stimulation. The IL-1β and IL-10 levels were significantly higher and were stimulated via dectin-1. Phagocytosis and stimulation of neutrophil extracellular traps by human granulocytes were increased in highly *GP70*-silenced strains. Furthermore, these mutants showed virulence attenuation in the invertebrate model *Galleria mellonella*. Our results demonstrate that Gp70 is a versatile protein with adhesin properties, is responsible for the activity of 3-carboxy-*cis*,*cis*-muconate cyclase, and is relevant for the *S. schenckii*–host interaction.

## 1. Introduction

*Sporothrix schenckii* is a hypha-producer fungus that belongs to the pathogenic clade of the *Sporothrix* genus [1,2]. This species, along with *Sporothrix brasiliensis* and *Sporothrix globosa*, are the main etiological agents of sporotrichosis, a cutaneous and subcutaneous mycosis that affects humans and other mammals, in particular, domestic species, such as cats and dogs [3,4]. The disease is distributed worldwide, but epidemic areas of *S. schenckii*-caused sporotrichosis have been identified in Peru, Mexico, China, Japan, India, South Africa, Australia, Colombia, and Venezuela [5]. Different from opportunistic infections caused by *Candida albicans*, *Aspergillus fumigatus*, and *Cryptococcus neoformans*, which are associated with high mortality rates [6], most of the sporotrichosis cases are not life-threatening infections. Lymphocutaneous and fixed cutaneous infections are the most frequent sporotrichosis forms, the latter a disease autolimited by the host immunity that avoids fungal spread to deep-seated organs [7,8].

Fungal virulence is a complex process that involves the display of virulence factors and determinants by the pathogen. In addition, a permanent or temporally impaired immunity may contribute to the host’s susceptibility to the infection [9]. The presence of adhesins, hydrolytic enzymes, dimorphism, oxidative and nitrosative stress, thermotolerance, and the ability to evade the host immunity are the canonical factors behind fungal virulence and are thought to be used by *S. schenckii* [10,11]. The cell wall is an external organelle that fulfills different functions in fungi, including protection from changes in the extracellular environment, providing shape to cells, control of the intracellular pressure, and working as a molecular scaffold to display different molecules at the cell surface, including virulence factors [12]. Adhesins are one of the virulence factors abundantly studied in *S. schenckii*. There are studies linking cell wall glycoproteins of yeast-like cells with the adhesion to epithelial cells [13] and components of the extracellular matrix (type II collagen, fibronectin, fibrinogen, and laminin) [14,15]. Immunodetection approaches identified a 70-kDa glycoprotein (Gp70) as one of the main *S. schenckii* adhesins that bind to fibronectin [16]. Moreover, anti-Gp70 antibodies reduced *S. schenckii* adhesion to mouse tail dermis [17].

Gp70 is a highly glycosylated 3-carboxy-*cis*,*cis*-muconate cyclase found in the cell walls of *S. schenckii*, *S. globosa*, and *S. brasiliensis* [18,19]. The glycosylation associated with this protein is prominent, as the peptide backbone is predicted to be 43 kDa, contrasting with the native form, which is a complex of at least six glycoforms, ranging from 70 to 60 kDa [18,19]. In addition to the cell wall-associated protein, Gp70 has been also identified in the extracellular compartment, suggesting this is also a secreted glycoprotein [20]. The role of this glycoprotein as an adhesin was challenged when Gp70 levels were measured in *Sporothrix* cells with different virulence degrees. It was found that a higher amount of this protein was associated with low virulence strains, while poor Gp70 detection was observed in highly virulent strains of *S. brasiliensis* [20]. Thus, currently, this protein is considered both an adhesin and an immunogenic cell wall component that helps the host immunity to establish a protective anti-*Sporothrix* immune response [20]. The latter is supported by the fact that a monoclonal anti-Gp70 antibody is capable of controlling experimental sporotrichosis, indicating this antibody may have therapeutic applications [21,22]. Furthermore, it has been observed that the Gp70 peptide backbone, lacking modification through glycosylation, is enough to generate a protective anti-*Sporothrix* response based on Gp70 detection [19,23,24].

Here, to provide additional evidence about Gp70′s role in *S. schenckii* pathogenesis, we generated *GP70*-silenced mutants and characterized their phenotype, with particular emphasis on the ability to bind to extracellular matrix components and the *Sporothrix*–host interaction.

## 2. Materials and Methods

### 2.1. Strains and Culturing Conditions

*S. schenckii* ATCC MYA-4821 was used in this study. This is a reference strain [20] whose genome has been already sequenced [25] and previously used for gene silencing. Cells were propagated in YPD broth, pH 4.5 (1% [*w*/*v*] yeast extract, 2% [*w*/*v*] gelatin peptone, and 3% [*w*/*v*] dextrose), and incubated at 28 °C and 120 rpm for 48 h. *Sporothrix* cells containing the binary vector were selected on YPD plates, pH 4.5, containing 400 mg mL^−1^ hygromycin B (GoldBio, St Louis, MO, USA) [26]. Conidia, filament growth, and dimorphism stimulation measurements for yeast-like cells were performed in YPD, as previously standardized [27]. The growing conditions of the medium for yeast-like cells were adjusted to pH 7.8 and incubated at 37 °C and 120 rpm for four days [27]. When required, cells were incubated at 60 °C for 2 h for heat inactivation [27]. Loss of cell viability was confirmed by incubating on YPD plates, pH 4.5, at 28 °C for 5 days [20].

For the case of bacteria, *Agrobacterium tumefaciens* AGL-1 was used in this work and was grown in Luria–Bertani broth (0.5 [*w*/*v*] yeast extract, 1% [*w*/*v*] gelatin peptone, and 1% [*w*/*v*] NaCl) at 28 °C and 120 rpm for 24 h. When required, the medium was supplemented with 100 μg mL^−1^ ampicillin and 100 μg mL^−1^ kanamycin for bacteria selection.

### 2.2. Molecular Techniques

To generate the binary plasmid for *GP70* silencing, we used the pBGgHg backbone [28]. The sense and antisense specific fragments were synthesized by PCR using the following primer pairs that align in the same 295 pb region closer to the 5′ end of the *GP70* open reading frame (GenBank accession code KF275147.1): 5′-CTCGAGTTCCTGAGCAGCCTGGACGG and 5′-AAGCTTAGGTCTCCGTCAGGTTGGG, for sense fragment, and 5′-AGGCCTTTCCTGAGCAGCCTGGACGG and 5′-AGATCTAGGTCTCCGTCAGGTTGGG, for antisense fragment. This region was free of restriction sites of interest and predicted intronic regions. The sense fragment was cloned into the pSilent-1 [29] XhoI and HindIII sites, while the antisense fragment was inserted into the StuI and BglII sites [26], generating pSilent-1-GP70. This plasmid worked as a template to amplify by PCR a 2774 bp amplicon using the primer pair 5′-CTGCAGATGCCAGTTGTTCCCAGTGATC and 5′-GAGCTCCCTCTAAACAAGTGTACCTGTGCATT, which spans from the *Aspergillus nidulans trpC* promoter contained in pSilent-1 to the *A. nidulans trpC* terminator, including the multi-cloning site where the sense and antisense regions were cloned [29]. This amplicon was cloned into the pBGgHg PstI and SacI sites, obtaining pBGgHg-GP70. This construction was used in *Agrobacterium*-mediated *Sporothrix* transformation, as described elsewhere [26]. Transformations of conidia were performed in the presence of 200 μM acetosyringone (Sigma-Aldrich, San Luis, MO, USA), and fungal cells were selected on YPD, pH 4.5, supplemented with 400 mg mL^−1^ hygromycin B and 200 μM of cefotaxime (Sigma-Aldrich), and incubated at 28 °C for 72 h [26]. For transformed nuclei enrichment, five monoconidial passages were made in YPD, pH 4.5, with 400 mg mL^−1^ hygromycin B, plus three dimorphism induction events in YPD, pH 7.8, with 400 mg mL^−1^ hygromycin B [26]. The insertion of the exogenous plasmid into the *S. schenckii* genome was confirmed by PCR using the primer pair 5′-GGCGACCTCGTATTGGGAATC and 5′-CTATTCCTTTGCCCTCGGACGAG.

For gene expression analyses, total RNA was extracted using standardized methods, cDNA was synthesized using the oligo(dT)20 primer [30], purified by chromatography, and the concentration was quantified in a NanoDrop 2000 (Thermo Fisher Scientific, Waltham, MA, USA). The quantitative PCR (qPCR) reactions contained cDNA, SYBR Green PCR Master Mix, and the primer pair 5′-TTCCTGAGCAGCCTGGACGG and 5′-AGGTCTCCGTCAGGTTGGG, and they were performed in a thermocycler StepOne Plus (Life Technologies, Carlsbad, CA, USA). The amplified region corresponds to the *GP70* sense region cloned into pBGgHg-GP70. For data normalization, amplification of the L6 gene was performed using the primer pair 5′-ATTGCGACATCAGAGAAGG and 5′-TCGACCTTCTTGATGTTGG [30]. The amplification of this gene from strain ATCC MYA-4821 was defined as the reference condition. Data were analyzed in the StepOne software V 2.2 (Life Technologies) using the 2^−∆∆Ct^ method [31]. To assess the number of binary plasmid insertional events, we followed the same strategy but using genomic DNA [26]. To assess the expression of *MKC1* (*SLT2*), the putative ortholog in *S. schenckii* was predicted (GenBank accession code XM_016735816), and RT-qPCR reactions were performed with the primer pair 5′-GCGGCCAAACAACTTCAATG and 5′-ATCGCAAATCTTGAGCTCGC.

### 2.3. 3-Carboxy-cis,cis-Muconate Cyclase Activity Assays

The assays were conducted as previously described [19]. Briefly, yeast-like cells were grown in YPD, pH 7.8, for 4 days at 37 °C; then, cells were pelleted by centrifuging, washed with citrate buffer, pH 6.0, and disrupted with glass beads in an MSK cell homogenizer (Braun, Melsungen, Germany). Cell debris was removed from the homogenate by centrifuging at 3000× *g* for 15 min at 4 °C. The supernatant was saved and used for enzyme activity assays. The 3-carboxy-*cis*,*cis*-muconate cyclase activity was measured using 50 µg of protein in 0.1 M citrate buffer, pH 6.8. The absorbance at 260 nm was measured, then 100 µL of 10 mM 3-carboxy-*cis*-*cis*-muconate was included, and the reaction mixtures were incubated for 5 min at 30 °C. Then, absorbance at 260 nm was measured again. The shift in absorbance at 260 nm was associated with the generation of the product 3-carboxymuconolactone [19,32]. The 3-carboxy-*cis*-*cis*-muconate was synthesized as described [19], and specific activity was defined as Δ_260nm_ min^−1^ mg protein^−1^.

### 2.4. Adhesion Assays

An ELISA-based method was performed as described [14,33]. Aliquots containing 0.05% (*w*/*v*) PBS-Tween 20 and 1 µg of the following extracellular matrix components were used to coat wells of Nunc MaxiSorp™ flat-bottom 96-well microplates (Sigma-Aldrich): laminin, elastin, fibrinogen, recombinant fibronectin, recombinant thrombospondin-1, type I collagen (all from human source, Sigma-Aldrich), or bovine type II collagen (Sigma-Aldrich). Plates were incubated for 3 h at room temperature, then incubated overnight at 4 °C with 1% (*w*/*v*) bovine serum albumin (BSA) in PBS, and washed three times with 0.05% (*w*/*v*) PBS-Tween 20.

The adhesion assays were performed with 5 × 10^6^ yeast-like cells suspended in 100 µL of PBS. After adding cells to the wells, plates were incubated for 1 h at 37 °C, washed three times with 0.05% (*w*/*v*) PBS-Tween 20, 100 µL of polyclonal anti-rHsp60 [33] at a working dilution of 1:3000 was added, and the plates were incubated for 2 h at room temperature. Plates were washed with 0.05% (*w*/*v*) PBS-Tween 20, 100 µL of goat anti-rabbit IgG-peroxidase antibody (Sigma-Aldrich) was added at a working dilution of 1:5000, and the plates were further incubated for 2 h at room temperature. The antibody–antigen interaction was revealed by adding 0.1 mg mL^−1^ 2,2′-azino-bis(3-ethylbenzothiazoline-6-sulfonic acid) diammonium salt and 0.006% (*v*/*v*) hydrogen peroxide and incubating at room temperature for 20 min. The reaction was stopped with 2 N sulfuric acid. Plates were read at 450 nm in a Varioskan LUX Multimode Microplate Reader (Thermo Fisher Scientific). When indicated, fungal cells were incubated with polyclonal antibodies against rGp70 at a working dilution of 1:3000 for 60 min at 37 °C, before being used in the adhesion assays.

### 2.5. Cell Wall Analysis

Yeast-like cells were grown for 4 days at 37 °C in YPD, pH 7.8, and disrupted with glass beads in an MSK cell homogenizer. Homogenates were centrifuged, and cell walls were pelleted and cleaned by washing with hot SDS, β-mercaptoethanol, and NaCl [34]. Aliquots containing 5 mg of cell wall were hydrolyzed with 2 M trifluoroacetic acid; the acid evaporated, the digested material was resuspended in deionized water, and the product was analyzed by high-performance anion-exchange chromatography with pulsed amperometric detection (HPAEC-PAD), as reported [27]. For protein quantification, the cleaned walls were suspended in 1 N NaOH, boiled for 30 min, and neutralized with 1N HCl, and the protein content was determined with the Pierce BCA Protein Assay (Thermo Fisher Scientific) [35]. The cell wall porosity was analyzed by the relative ability of polycations to interact with the plasma membrane [36]. A 1 mL aliquot of a yeast-like cell suspension, containing 1 × 10^8^ cells, was centrifuged to pellet cells; these were suspended in 10 mM Tris-HCl, pH 7.4 (buffer A), buffer A plus 30 μg mL^−1^ poly-L-lysine (MW 30–70 kDa, Sigma-Aldrich), or buffer A plus 30 μg mL^−1^ diethyl aminoethyl-dextran (MW 500 kDa, Sigma-Aldrich) and incubated for 30 min at 28 °C under gentle shaking [36]. Then, preparations were centrifuged to form pellets, and the supernatants were saved and used to measure the absorbance at 260 nm [36]. The readings obtained with poly-L-lysine are considered 100% porosity, and the results are given as the relative porosity observed with diethyl aminoethyl-dextran [36]. The exposure of β-1,3-glucan and chitin at the cell wall surface was analyzed with fluoresce-label lectins, as reported [35,37]. To label chitin, yeast-like cells and 0.5 mg mL^−1^ fluorescein isothiocyanate conjugated wheat germ agglutinin (Sigma-Aldrich) were incubated for 60 min at room temperature, while for β-1,3-glucan labeling, cells were incubated with 5 μg mL^−1^ IgG Fc-dectin-1 chimera [38] for 40 min at room temperature and then incubated under the same conditions with 1 μg mL^−1^ donkey anti-Fc IgG-FITC (Sigma-Aldrich). Cell preparations were inspected under fluorescence microscopy with a Zeiss Axioscope-40 microscope (Carl Zeiss AG, Jena, Germany) and an Axiocam MRc camera. Three hundred cells per sample were analyzed, acquiring the median fluorescence per cell, as described [27]. Heat-inactivated yeast-like cells were also labeled as described, and the associated fluorescence was regarded as 100% polysaccharide exposure at the cell wall surface.

To assess changes in the negative net charge of the wall, cells were stained with Alcian blue (Sigma-Aldrich), and the amount of dye bound was quantified. Briefly, aliquots of 1 mL containing yeast-like cells at an O.D._600nm_ of 0.2 were pelleted, suspended in 1 mL of 30 μg mL^−1^ Alcian blue in 0.02 M HCl, pH 3.0, and incubated for 10 min. Cells were pelleted, and the supernatant was used to measure the amount of unbound dye, as reported [39].

### 2.6. Quantification of Cell Wall N-Linked and O-Linked Glycans

Yeast-like cells were adjusted to 1 × 10^9^ cells mL^−1^, suspended in 3 mM NaOAc and 25 U endoglycosidase H (New England Biolabs, Ipswich, MA, USA), and incubated at 37 °C for 24 h [26]. Then, the preparations were neutralized, the cells were pelleted, and the soluble *N*-linked glycans were saved and kept at −20 °C until use. For *O*-linked glycans, the same amount of cells were suspended in 0.1 N NaOH and incubated overnight at room temperature with gentle orbital shaking [26]. In both cases, glycans were acid-hydrolyzed as previously described, and the sugar content was analyzed by HPAEC-PAD [26].

### 2.7. Ethics Statement

Human blood samples were collected from healthy donor volunteers after informed consent was signed. This study was conducted following the Declaration of Helsinki, and the Ethics Committee of Universidad de Guanajuato authorized this study (reference CEPIUG-P48-2022).

### 2.8. Analysis of the Fungus–Human Mononuclear Cell Interaction

Human peripheral blood mononuclear cells (PBMCs) were isolated from venous blood samples by differential centrifugation in Histopaque-1077 (Sigma-Aldrich), as described [40]. Aliquots containing 100 μL 1 × 10^5^ yeast-like cells and 100 μL 5 × 10^5^ PBMCs in RPMI 1640 Dutch modification (enriched with 2 mM glutamine, 0.1 mM pyruvate, and 0.05 mg mL^−1^ gentamycin; all reagents from Sigma-Aldrich) were placed in round-bottom 96-well microplates and incubated for 24 h at 37 °C with 5% (*v*/*v*) CO_2_. Then, plates were centrifuged for 10 min at 1800× *g* at 4 °C, and supernatants were saved and used for cytokine quantification [27]. Standard ABTS ELISA Development kits (Peprotech, Cranbury, NJ, USA) were used to determine the levels of human tumor necrosis factor-alpha (TNFα), interleukin 6 (IL-6), interleukin 1β (IL-1β), and interleukin 10 (IL-10). Mock wells where PBMCs were incubated only with a culturing medium were used as controls.

To assess the contribution of some immune receptors during the *Sporothrix*–PBMC interaction, the immune cells were preincubated for 1 h at 37 °C with 200 μg mL^−1^ laminarin (Sigma-Aldrich) [27], 10 μg mL^−1^ anti-mannose receptor (MR) (Thermo-Fisher Scientific, MA5-44033), 10 μg mL^−1^ anti-TLR4 antibody (Santa Cruz Biotechnology, Dallas, TX, USA sc-293072), 10 μg mL^−1^ anti-TLR2 antibody (Thermo-Fisher Scientific, Waltham, MA, USA 16-9922-82), or 10 μg mL^−1^ anti-CD11b antibody (CR3, Thermo Fisher Scientific, MA5-16528). All the antibody solutions were prepared with 5 μg mL^−1^ polymyxin B (Sigma-Aldrich) to neutralize any trace of bacterial lipopolysaccharide [41]. As controls, isotype-matched irrelevant antibodies were used during the preincubation stage. Antibodies were IgG1 antibody (10 μg mL^−1^, Santa Cruz Biotechnology, Cat. No. sc-52003, used as a control in experiments where TLR4 and MR were blocked), 10 μg mL^−1^ IgG2aκ antibody (Thermo-Fisher Scientific, 14-4724-85, to control experiments where TLR2 was blocked), and 10 μg mL^−1^ IgG2 antibody (R&D, Minneapolis, MN, USA, Cat. No. MAB9794, to control experiments were CD11b was blocked) [27,42].

### 2.9. Analysis of Phagocytosis by Human Granulocytes

The granulocytes/red blood cells phase was obtained from the density gradient separation of human blood described in Section 2.8. To disrupt erythrocytes, cells were suspended in 50 mL 154.4 mM ammonium chloride, 10 mM potassium bicarbonate, and 97.3 mM EDTA tetrasodium salt [43]. Granulocytes were suspended in RPMI-1640 Dutch modification and inspected under bright light microscopy to assess degranulation and to perform differential counting. All cell preparations showed 97 ± 1.2% of neutrophils.

For phagocytosis analysis, fungal cells were incubated with 1 mg mL^−1^ Acridine Orange (Sigma-Aldrich) for 30 min at room temperature, washed twice with cold PBS, and adjusted to 3 × 10^7^ cells mL^−1^ [44]. The innate immune cell–yeast-like cell interactions were performed in 6-well plates using an immune cell/fungus ratio of 1:6 in 800 µL of DMEM medium (Sigma-Aldrich). The plates were incubated for 2 h at 37 °C and 5% (*v*/*v*) CO_2_; then, immune cells were detached with chilled PBS and incubated with 1.25 mg mL^−1^ Trypan Blue [44]. Phagocytosis was analyzed by flow cytometry in an FACSCanto II system (Becton Dickinson, Franklin Lakes, NJ, USA). Samples were analyzed with the FL1 and FL2 channels, previously calibrated with non-stained human cells. From each sample, a total of 50,000 events were collected. The results correspond to events gated in the R7 quadrant, corresponding to cells mainly fluorescing in red, i.e., cells in the late stage of phagocytosis [26]. The preincubation of human granulocytes with either laminarin or any of the anti-receptor antibodies was performed essentially as described in Section 2.8.

### 2.10. Quantification of Neutrophils Extracellular Traps

Neutrophil extracellular traps (NETs) were formed as previously described [45]. Ninety six-well plates were coated with 1% bovine serum albumin, and aliquots of 175 µL of human granulocytes at 4 × 10^7^ cells mL^−1^ in RPMI 1640 were placed into each well and incubated for 30 min at 37 °C and 5% (*v*/*v*) CO_2_. Then, 25 µL of yeast-like cells at 4 × 10^8^ cells mL^−1^ were added to the wells and further incubated for 4 h at 37 °C and 5% (*v*/*v*) CO_2_. Then, plates were centrifuged for 10 min at 1800× *g* and 4 °C, and the supernatant was collected and used to quantify nucleic acids by spectrophotometry at 260 nm in a NanoDrop One (Thermo Fisher Scientific). As a control, mock interactions containing only human cells and PBS were included.

### 2.11. Virulence Assays in Galleria mellonella

Assays were performed with larvae from a previously established local colony [46] and fed ad libitum with a conventional diet for this species [47]. The inclusion criteria for inoculation were a length between 1.2 and 1.5 cm, active behavior, and no apparent melanization. Fungal challenges included 1 × 10^5^ yeast-like cells in 10 μL of PBS, and these were injected in the last left pro-leg with a Hamilton syringe and a 26-gauge needle, as reported [46]. Animals were inspected for 14 days under hydration ad libitum with chopped apple and a 37 °C temperature. Larvae were considered dead when irritability to external stimuli was lost and the body surface was extensively melanized [46]. Groups of 30 larvae were used to analyze the virulence of each of the *Sporothrix* strains used in this work. One additional group inoculated only with PBS was included as a control. Colony-forming units, hemocyte concentration, phenoloxidase activity, melanin production, and cytotoxicity were measured, as previously reported [19,47], using anticoagulated hemolymph. Phenoloxidase activity was assayed using 20 mM 3,4-dihydroxyDL-phenylalanine (Sigma-Aldrich), whilst cytotoxicity was quantified with the Pierce LDH Cytotoxicity Assay (Thermo Fisher Scientific).

### 2.12. Statistical Analysis

Analyses were performed on GraphPad Prism 6 software (Version 6.07) using the Mann–Whitney U and Kruskal–Wallis tests for results obtained with human cells and larvae. Other results were analyzed with the parametric Student’s test. In all cases, the significance level was set at *p* < 0.05.

## 3. Results

### 3.1. Silencing of Sporothrix schenckii GP70

The *A. tumefaciens*-mediated *Sporothrix* transformation with the binary plasmid pBGgHg has been previously used to silence genes in *S. schenckii* [26,48]. Thus, a similar strategy was used here, transforming fungal cells with pBGgHg-GP70. Transformed cells were selected in YPD supplemented with 400 mg mL^−1^ hygromycin B, and monoconidial passages were made to enrich transformed nuclei, as described in the Section 2. Then, PCR reactions to assess the presence of the binary plasmid were performed, amplifying the *Escherichia coli hph* gene contained within pBGgHg. Only PCR-positive colonies were selected for analysis of *GP70* gene expression by RT-qPCR. To assess the possible contribution of the binary plasmid to the phenotypes associated with *GP70* silencing, strain ATCC MYA-4821 (referred to hereafter as wild-type, WT) was also transformed with the empty vector, and two random PCR-positive colonies were also included in both the molecular and phenotypical characterization. The gene expression analysis showed mutant strains with different levels of *GP70* silencing (Table 1). Strains HSS39 and HSS40, transformed with the empty vector, showed a similar *GP70* expression to the WT strain and, therefore, were used as transformation controls. Strains HSS41-HSS46 were obtained after transformation with pBGgHg-GP70 and, based on the *GP70* expression, were classified as strains with intermediate *GP70* silencing (HSS41, HSS42, and HSS43) or highly *GP70*-silenced strains (HSS44, HSS45, and HSS46; see Table 1).

Next, we assessed the number of integrative events of the binary vector within the *S. schenckii* genome, as multiple and random integrative events of this plasmid have been previously reported during *A. tumefaciens*-mediated *Sporothrix* transformation [26]. This was assessed by qPCR, amplifying the *GP70* fragment cloned into the binary vector. Since the *S. schenckii* genome is haploid [49], it is expected to amplify one copy of this fragment from the WT strain and three copies of this fragment in mutants harboring one copy of the binary plasmid: one from the native locus, one from the sense fragment, and one from the antisense region. Thus, the amplification of more than four copies of this fragment would suggest the presence of more than one copy of the binary plasmid within the fungal genome. The results shown in Table 1 indicated that all the *GP70*-silenced strains showed three copies of this *GP70* fragment, while the WT and the control strains showed only one copy. Thus, all the transformed strains have one binary plasmid insertional event. All the silenced mutants showed no defects in cell growth, generating yeast-like cells, hyphae, and conidia as the WT and control strains. The average doubling times were similar to the WT and control strains (hypha 3.2 ± 0.6 h vs. 3.3 ± 0.5 h, and yeast-like cells 7.8 ± 0.4 h vs. 8.2 ± 0.3 h, for WT and silenced mutants, respectively).

### 3.2. Silencing of Sporothrix schenckii GP70 Affected the 3-Carboxy-cis,cis-Muconate Cyclase Activity and Cell Adhesion

Next, to confirm the silenced mutants have reduced levels of Gp70, we measured the Gp70 enzyme activity, which has been defined as 3-carboxy-*cis*,*cis*-muconate cyclase [18,19]. This was measured by the change in the absorbance at 260 nm of the substrate 3-carboxy-*cis*,*cis*-muconate. The WT and control strains showed similar levels of enzyme activity, and this was significantly reduced in all of the six silenced strains (Table 1). Strains HSS41, HSS42, and HSS43 showed higher enzyme activity levels than strains HSS44, HSS45, and HSS46, confirming that the former group has intermediate silencing levels and enzyme activity, whereas the latter has strong silencing levels and traces of enzyme activity (Table 1). Control reactions where heat-inactivated homogenates were used gave threshold levels of absorbance change (5.4 × 10^−3^ ± 3.2 × 10^−3^ Δ_260nm_ min^−1^ mg protein^−1^, on average for all tested strains). Similarly, mock reactions where water was included instead of the substrate gave minimal values (3.1 × 10^−3^ ± 1.1 × 10^−3^ Δ_260nm_ min^−1^ mg protein^−1^).

**Figure 1:**
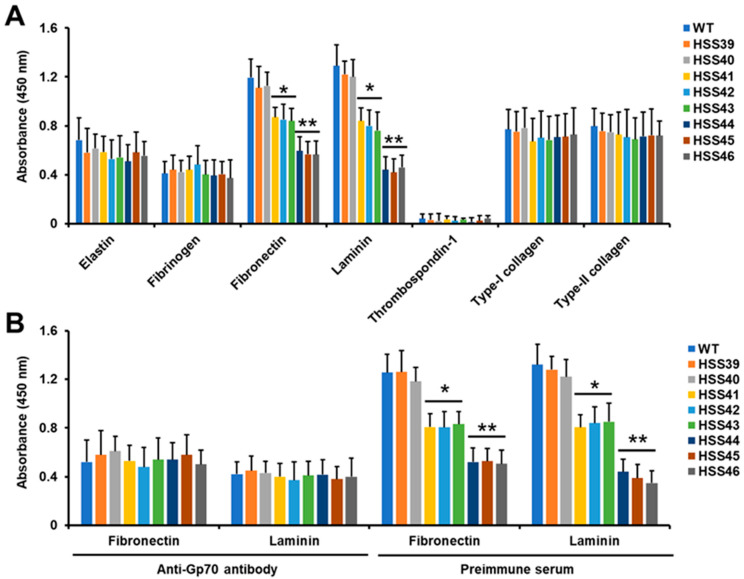
The *Sporothrix schenckii GP70*-silenced mutants showed reduced adhesion to extracellular matrix proteins. In (**A**), proteins were immobilized in 96-well plates, yeast-like cells were added, and the plates were incubated for 1 h at 37 °C. Plates were washed to remove non-adherent cells, and bound yeast-like cells were detected with anti-rHsp60 antibodies, as described in the Section 2. WT, strain ATCC MYA-4821. (**B**) followed the same steps as panel A, but yeast-like cells were preincubated with anti-Gp70 antibody or with preimmune serum before the adhesion assays. Results are means ± SD of three independent experiments performed in duplicate. * *p* < 0.05 when compared to WT or strains HSS39 and HSS40. ** *p* < 0.05 when compared to strains HSS41, HSS42, and HSS43.

As mentioned, previous reports have suggested that Gp70 has adhesive properties [16,17]; therefore, we explored whether this virulence trait was affected in the silenced mutant strains. We tested a panel of different extracellular matrix components, for which *S. schenckii* yeast-like cells have previously shown adhesion, except for thrombospondin-1 [14,33]. The adhesion to human elastin, fibrinogen, thrombospondin-1, type-I collagen, and bovine type-II collagen was similar for WT, control, and silenced strains, indicating no participation of Gp70 in the adhesion to these proteins (Figure 1A). However, in the case of human fibronectin and laminin, the silenced strains showed a significant decrement in the ability to bind these proteins (Figure 1A). Strains with high levels of *GP70* silencing (HSS44, HSS45, and HSS46) showed a major decrement in the ability to bind both proteins, while strains HSS41, HSS42, and HSS43 showed intermediate levels of binding to these proteins, being lower when compared to WT and control strains but higher when compared to the other silenced strains (Figure 1A). Since these results suggested that Gp70 was involved in adhesion to human fibronectin and laminin, we next performed adhesion experiments with yeast-like cells preincubated with either anti-Gp70 polyclonal antibodies, previously generated by our group [19], or preimmune serum. When anti-Gp70-coated yeast-like cells were used to interact with human fibrinogen or laminin, a poor binding ability was observed for the WT strain, which was similar to the control strains (Figure 1B). No changes in the ability to bind both human proteins were observed with the silenced strains preincubated with anti-Gp70 antibodies (Figure 1B). The control assays with preimmune serum showed the same adhesion profile observed in Figure 1A, with high levels of adhesion associated with the WT and control strains, intermediate levels for HSS41, HSS42, and HSS43, and the lowest adhesion level with the strains HSS44, HSS45, and HSS46 (Figure 1B). Control experiments only with the secondary antibody showed threshold readings. Therefore, these results indicate that Gp70 is associated with adhesion to human fibronectin and laminin.

### 3.3. Silencing of Sporothrix schenckii GP70 Affected the Cell Wall Composition and Organization

**Figure 2:**
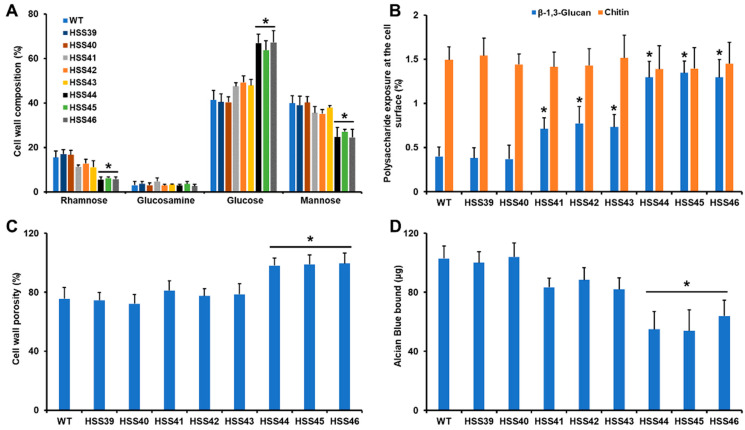
Cell wall analysis of *Sporothrix schenckii* wild-type, control, and *GP70*-silenced mutant strains. In (**A**), the cell walls were incubated with trifluoroacetic acid, hydrolyzing oligosaccharides and polysaccharides. The resulting monosaccharides were separated and quantified by high-performance anion-exchange chromatography with pulsed amperometric detection. In (**B**), yeast-like cells were incubated with specific lectins to label either chitin or β-1,3-glucan, and the fluorescence of 300 cells was quantified. Lectins were fluorescein isothiocyanate conjugated wheat germ agglutinin or IgG Fc-dectin-1 chimera and anti-Fc IgG-fluorescein isothiocyanate for chitin or β-1,3-glucan labeling, respectively. In (**C**), yeast-like cells were incubated with diethyl aminoethyl-dextran, and the released material absorbing at 260 nm was quantified. The readings at 260 nm obtained with poly-L-lysine were used for data normalization and regarded as 100%. In (**D**), yeast-like cells were incubated with 30 µg of Alcian blue and the quantification of the unbound dye was used to estimate the dye bound to cells. Data were normalized to dye bound by cells at an OD_600nm_ = 1.0. Data are represented as mean ± SD of three independent experiments performed in duplicate. * *p* < 0.05 when compared to WT, HSS39, or HSS40 strains.

Next, we assessed the impact of *GP70* silencing on the cell wall composition. Cell walls were incubated in 2M trifluoroacetic acid, and the polysaccharides and oligosaccharides were hydrolyzed to monosaccharides. Under these conditions, β-glucans released glucose, chitin was broken down in the glucosamine units, whilst *O*-linked and *N*-linked glycans attached to glycoproteins released mannose and rhamnose [27]. The six mutant strains, along with the control and WT strains, showed similar levels of chitin, but β-glucans and mannose- and rhamnose-containing glycans were different (Figure 2A). In the case of β-glucans, these were significantly increased in the highly silenced mutants (HSS44, HSS45, and HSS46; Figure 2A). Even though the other silenced mutants showed an increment in the content of this polysaccharide, this was not statistically significant (Figure 2A). For the case of rhamnose and mannose levels, these were significantly reduced in the highly *GP70*-silenced mutant strains but not in the mutants HSS41, HSS42, or HSS43 (Figure 2A).

It has been previously demonstrated that the structural polysaccharides β-1,3-glucan and chitin are mainly found in the inner layer of the cell wall [26,27]. We hypothesized that the increment in β-glucan levels in some of the *GP70*-silenced strains may have an impact on the distribution of this wall polysaccharide. Using fluorescent-labeled lectins that bind to chitin and β-1,3-glucan, we assessed the levels of these polysaccharides on the cell wall surface [35,37]. The results indicated that there were no changes in the levels of chitin exposed at the cell surface, but β-1,3-glucan was more exposed on the wall surface of the *GP70*-silenced mutant strains (Figure 2A). The highly silenced strains showed increased exposure levels when compared to strains with intermediate silencing levels (Figure 2B).

The cell wall porosity is a sensitive parameter that is affected when fungal cells have defects in the cell wall composition [48], while the ability of cells to bind Alcian blue is a useful parameter to assess changes in the net negative charge of the cell wall [39]. The control and WT strains showed similar cell wall porosity and ability to bind Alcian blue, and these parameters were similar to those observed with the strains with intermediate levels of *GP70* silencing (Figure 2C,D). The strains HSS44, HSS45, and HSS46 showed a significant increment in cell wall porosity and a decrement in the ability to bind Alcian blue, in line with the observed changes in cell wall composition (Figure 2C,D).

**Figure 3:**
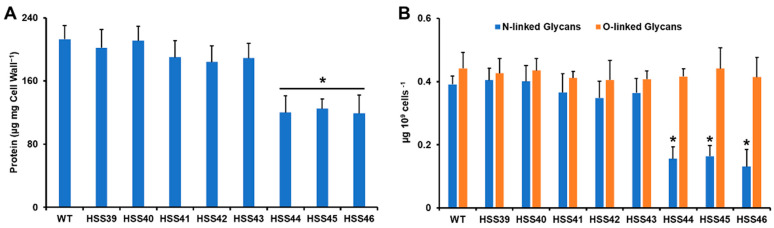
The cell wall protein and *N*-linked glycan content are affected in the *Sporothrix schenckii GP70*-silenced mutant strains. In (**A**), cell walls were alkali-hydrolyzed, and the protein content was measured and normalized to one mg of cell wall dry weight. In (**B**), yeast-like cells were incubated either with endoglycosidase H or β-eliminated to remove *N*-linked or *O*-linked glycans, respectively. The released oligosaccharides were measured and data normalized to 10^9^ yeast-like cells. Data are shown as mean ± SD of three independent experiments performed in duplicate. * *p* < 0.05 when compared to WT, HSS39, or HSS40 cells.

The decrement in rhamnose- and mannose-based glycans observed in the *GP70*-silenced mutants HSS44, HSS45, and HSS46 may be related to the reduction in the Gp70 content at the cell wall. Therefore, the cell wall protein content was measured. The results showed that WT, control, and mutants with intermediate silencing levels showed similar cell wall protein content, but strains with high *GP70* silencing showed a significant reduction in cell wall protein (Figure 3A). We have previously demonstrated that most of the glycans attached to Gp70 are *N*-linked glycans [19]. Thus, we hypothesized that due to the significant decrement of cell wall protein, the highly *GP70*-silenced mutant could show reduced levels of *N*-linked glycans. The cell walls were treated either with endoglycosidase H or β-eliminated to remove *N*-linked glycans or *O*-linked glycans, respectively [26,27,48]. The results showed that none of the analyzed strains had changes in the cell wall *O*-linked glycan content (Figure 3B). However, strains HSS44, HSS45, and HSS46, but not the other silenced strains, showed a significant reduction in the cell wall *N*-linked glycan content. Collectively, these data indicated that *GP70* silencing affected the cell wall composition and organization. Changes in cell wall components usually activate the cell wall integrity pathway in organisms such as *C. albicans* and *Saccharomyces cerevisiae* [50,51]. This involves the activation of Pkc1 and Mck1 and the compensatory increment in structural cell wall polysaccharides, such as β-glucans and chitin [51]. When the expression levels of the putative functional ortholog of *C. albicans MKC1* (*SLT2*) were quantified, we found no changes in gene expression in the control strains HSS39 and HSS40, but there was a 2.9 ± 0.9 fold change on average for strains HSS41-HSS43 and a 4.2 ± 1.4 fold change on average for strains HSS44-HSS46, suggesting activation of the cell wall integrity pathway in the silenced mutants.

### 3.4. The GP70 Silencing Affected the Human Innate Immune Cell–Sporothrix schenckii Interaction

**Figure 4:**
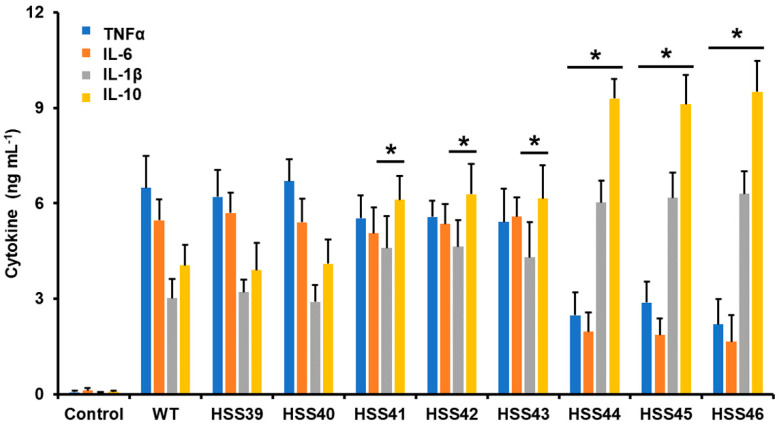
Cytokine production in human peripheral blood mononuclear cells stimulated by *Sporothrix schenckii* wild-type, control, and *GP70* silenced strains. Fungal and human cells were coincubated for 24 h at 37 °C, and the supernatants were collected and used to quantify cytokines by ELISA. Data are means ± SD obtained with samples from eight donors, assayed in duplicate wells. * *p* < 0.05 when compared to wild-type (WT) or control cells (HSS39 and HSS40).

Next, we assessed the impact of the *GP70* silencing on the interaction with human PBMCs. We used only yeast-like cells since this is the fungal morphology mainly found in infected tissues and, therefore, the most likely to be confronted by innate immune cells in an infection setting [52]. The WT control cells stimulated a previously reported cytokine pattern, with moderate TNFα levels, followed by IL-6, and IL-1β was the less stimulated cytokine [27]. The IL-10 stimulation was higher than IL-1β but not at the level observed for TNFα and IL-6 (Figure 4). This cytokine profile was mirrored by the control strains HSS39 and HSS40 (Figure 4). For the mutant strains with intermediate levels of *GP70* silencing (HSS41, HSS42, and HSS43), TNFα and IL-6 levels were similar to those stimulated by the WT and control strains, but IL-1β and IL-10 were significantly higher (Figure 4). The mutants with high levels of *GP70* silencing showed a similar trend in the IL-1β and IL-10 levels, higher than those associated with control and WT cells, and these were even significantly increased when compared with the groups of mutants with intermediate levels of silencing (*p* < 0.05 in all cases; Figure 4). However, in the case of this mutant group, they were incapable of stimulating TNFα and IL-6 to the same extent as the WT and control strains (Figure 4). These data indicated that the PBMCs–*S. schenckii* interaction was affected by the *GP70* silencing.

**Figure 5:**
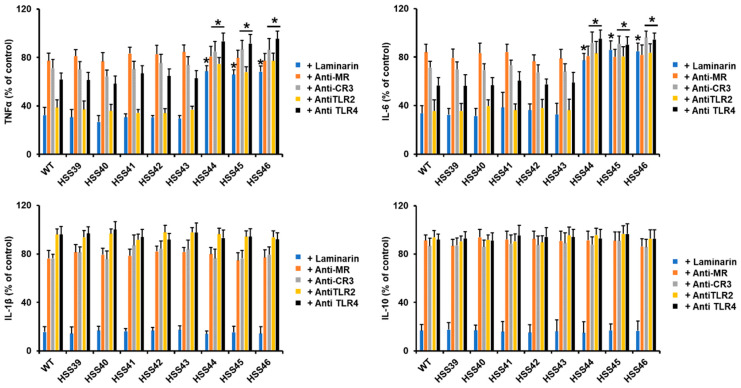
Cytokine production stimulated by *Sporothrix schenckii* wild-type, control, and *GP70*-silenced strains using human peripheral blood mononuclear cells preincubated with pattern recognition receptor antagonist. Human PBMCs were preincubated with laminarin or the respective antibody for 1 h at 37 °C and then were coincubated for 24 h at 37 °C with fungal cells. Plates were centrifuged, and the supernatants were collected and used to quantify cytokines by ELISA. Control interactions correspond to 100% and refer to interactions with no antagonist included, and the absolute values were similar to those observed in Figure 4. Data are means ± SD obtained with samples from eight donors, assayed in duplicate wells. * *p* < 0.05 when compared to wild-type (WT) or control cells (HSS39 and HSS40).

Next, we assessed the contribution of some pattern recognition receptors (PRRs) during the *Sporothrix*–PBMC interaction. We blocked dectin-1 with the antagonist laminarin [27] while blocking MR, CR3, TLR2, and TLR4 with monoclonal specific blocking antibodies [53,54]. The TNFα and IL-6 stimulation by the WT control strain was significantly affected by preincubation of human PBMCs with laminarin and anti-TLR2 and to a lesser extent by anti-TLR4 (Figure 5). The MR and CR3 had a discrete contribution to the stimulation of these cytokines (Figure 5). Similar profiles were observed with the control strains or the silenced mutants HSS41, HSS42, and HSS43 (Figure 5). However, for the case of the highly *GP70*-silenced mutants HSS44, HSS45, and HSS46, there was a reduction in the blocking effect observed with laminarin, anti-CR3, anti-TLR2, and anti-TLR4, indicating the ligands that stimulate both cytokines via these receptors are reduced in these fungal strains (Figure 5). When similar experiments were performed to analyze the contribution of these receptors for IL-1β and IL-10 stimulation, all the strains (WT, control, and *GP70*-silenced mutants) showed a similar trend. A strong influence of dectin-1 blocking on the production of both cytokines was observed, while the other receptors had a minor contribution (Figure 5). These results suggested that the production of both cytokines is via dectin-1. Control interactions with irrelevant isotype-matched antibodies gave cytokine levels similar to those observed in interactions with no antibody included.

**Figure 6:**
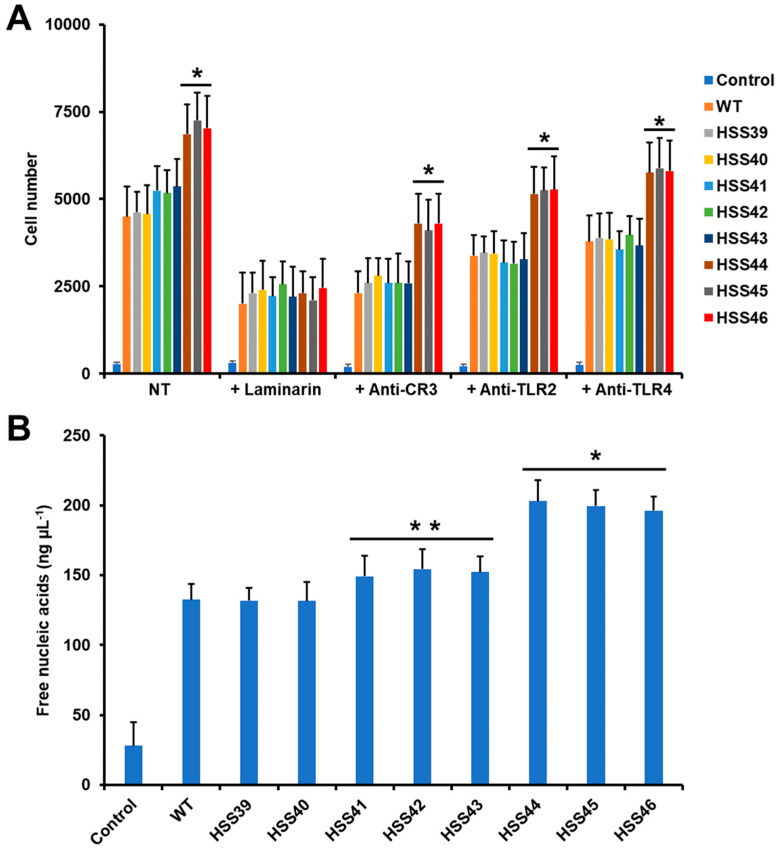
Phagocytosis and neutrophil extracellular trap analysis during the interaction of human granulocytes with *Sporothrix schenckii* wild-type, control, and GP70-silenced strains. In (**A**), yeast-like cells and human granulocytes we co-incubated for 2 h at 37 °C and 5% (*v*/*v*) CO_2_, and phagocytosis was analyzed by flow cytometry. Cells interacting with at least one yeast-like cell were included in the quantification. NT, immune cells were preincubated with PBS. Alternatively, the human cells were preincubated for 1 h at 37 °C with the corresponding antagonist of pattern recognition receptors and then used to asses phagocytosis. Control refers to human granulocytes interacting with PBS. In (**B**), the cell–cell interactions were incubated for 4 h at 37 °C and 5% (*v*/*v*) CO_2_, plates were centrifuged, and supernatants were collected. The extracellular traps were indirectly measured by quantifying the released nucleic acids. Control refers to human cells incubated with PBS. * *p* < 0.05 when compared to wild-type (WT) or control strains (HSS39 or HSS40). ** *p* < 0.05 when compared to strains HSS44, HSS45, or HSS46. Results are shown as mean ± SD with samples from eight donors analyzed in duplicate.

Next, we also assessed the effect of *GP70* silencing on the *S. schenckii*–human granulocyte interaction. We focused particularly on phagocytosis and the generation of NETs, as these immune responses are particularly relevant in the antifungal activity of these cells [55]. The WT and control strains were phagocytosed to the same extent, and even though the silenced strains HSS41, HSS42, and HSS43 tended to be more phagocytosed by granulocytes than the WT strain, these levels were not significant (*p* > 0.05; Figure 6A). The highly *GP70*-silenced mutant strains, HSS44, HSS45, and HSS46, showed a significant increment in fungal uptake when compared to the WT, control, and other silenced strains (Figure 6A). When the effect of some PRRs on the uptake by human granulocytes was assessed, we found that laminarin significantly reduced the fungal uptake in all the analyzed strains (Figure 6A). The blocking of CR3, TLR2, and TLR4 showed the same pattern: a reduced effect on fungal uptake, but this was more pronounced in the WT, control, and mutant strains with intermediate silencing levels (Figure 6A). The highly *GP70*-silenced strains also were negatively affected by the antagonists of these receptors, but the uptake levels were significantly higher when compared to the other analyzed strains (Figure 6A). Control assays with human cells incubated only with PBS gave threshold uptake values (Figure 6A). In addition, control interactions with irrelevant isotype-matched antibodies gave cytokine levels similar to those observed in interactions with no antibody included.

When NET production was evaluated, we found that nucleic acid release to the extracellular compartment was similar for the WT and control strains, but the silenced strains showed an increment in this parameter: the HSS41, HSS42, and HSS43 silenced strains showed a modest increment in the ability to stimulate NETs, but this was not significant when compared to WT or control cells (Figure 6B). On the contrary, the group composed of the silenced strains HSS44, HSS45, and HSS46 showed a significantly increased ability to stimulate NETs, which was different when compared to the WT, control, and intermediate *GP70*-silenced strains (Figure 6B). The control interaction, where granulocytes were incubated with PBS to assess non-specific NET formation, gave significantly low levels when compared to the cell–cell interactions (Figure 6B). Altogether, these data indicated that the *S. schenckii*–human granulocyte interaction is affected by *GP70* silencing.

### 3.5. Virulence Attenuation in the Sporothrix schenckii GP70-Silenced Mutant Strains

**Figure 7:**
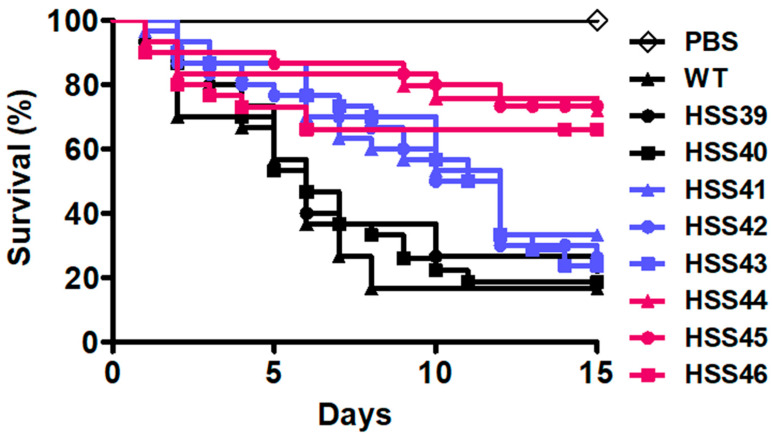
Virulence assays in *Galleria mellonella*. Groups containing 30 larvae were inoculated with each *Sporothrix schenckii* strain, and mortality was recorded daily for two weeks. WT refers to the parental strain ATTCC MYA-4821. PBS, larva group inoculated only with phosphate-buffered saline. Data are shown in Kaplan–Meier plots. Strains are grouped following a color code: the control group (black), strains with intermediate levels of *GP70* silencing (blue), and strains with high levels of *GP70* silencing (magenta). No significant differences were observed among group members, but the three groups were significantly different among themselves (*p* < 0.05).

Next, we analyzed the ability of *GP70*-silenced strains to kill *G. mellonella* larvae, a suitable system previously standardized to analyze *S. schenckii* virulence [46]. The WT strain showed the ability to kill 83.3 ± 6.2% of the larva population, with a median survival of 6.0 ± 0.7 days (Figure 7). The control strains HSS39 and HSS40 killed 76.6 ± 7.2% and 80.0 ± 5.7% of the animal population, respectively. Both strains showed a median survival of 6.0 ± 0.5 days (Figure 7). The strains with intermediate levels of *GP70* silencing, HSS41, HSS42, and HSS43, showed killing curves significantly different from the WT and control strains (*p* < 0.05; Figure 7). Strain HSS41 killed 66.6 ± 8.9% of animals with a median survival of 12.0 ± 0.8 days, while strains HSS42 and HSS43 both killed 73.3 ± 6.4% of larvae with a median survival of 11.5 ± 0.6 days. No significant differences were observed when these three strains were compared among themselves (Figure 7). For the highly *GP70*-silenced strains, HSS44 and HSS45 both killed 26.6 ± 8.8% of larvae, with a median survival of more than 15 days (Figure 7). Strain HSS46 killed 33.3 ± 7.6% of animals, with a median survival of more than 15 days (Figure 7). No differences were observed when the three strains were compared, but this group was significantly different from the curves generated by the strains with intermediate levels of *GP70* silencing, the WT, or control strains (Figure 7). A control group of animals injected only with PBS showed no mortality in the 15 days of observation (Figure 7). When fungal cells were collected from the hemolymph and the colony-forming units quantified, no changes were found in the fungal burden in the animal groups inoculated with the WT, control, or silenced strains (Table 2).

Additionally to the killing curves, some hemolymph parameters have been associated with virulence attenuation in *S. schenckii*. These include a reduction in hemocyte counts, cytotoxicity, measured as the cell-free lactate dehydrogenase, melanin formation, and phenoloxidase activity [26,48,56]. These four parameters were similar in the animal groups inoculated with the WT or control strains but tended to be significantly reduced in the six silenced strains under analysis (Table 2). The group with intermediate levels of *GP70* silencing showed significantly higher levels in the four parameters when compared with the group of highly silenced mutant strains (Table 2). The latter showed levels closer to those observed in the control group where animals were inoculated with PBS. Collectively, these data indicate that *GP70* silencing negatively affected virulence in *G. mellonella* larvae.

## 4. Discussion

The study of *S. schenckii* virulence factors is in the early stages when compared to other thoroughly studied fungi that cause infections in humans, such as *C. albicans*, *A. fumigatus*, and *C. neoformans*. The measurement of hydrolytic enzymes, adhesive properties, and biofilm stimulation has demonstrated these putative virulence factors are present in *S. schenckii*, but the identity of the genes involved in such cellular functions and their contribution to virulence and the interaction with the host are currently unknown. Melanin is the only virulence factor that has been studied by genetics and molecular biology. Albino mutants were generated using random mutagenesis or the new methodology CRISPR-Cas9, and it was found that this pigment is relevant during the interaction with the host and for resistance to oxygen- and nitrogen-derived oxidants and UV light-induced cell damage [57,58,59,60]. In the case of random mutagenesis, due to the nature of the technique, it is considered that the mutant strains do not display a clean phenotype, as additional mutations might be present in the genome, undermining the relevance of the phenotypes to understanding the role of melanin in *Sporothrix* pathogenesis. Thus, the generation of *GP70*-silenced strains establishes a step forward in the analysis of *S. schenckii* virulence factors and their interaction with the host. 

Gp70 is a controversial protein that was described more than a decade ago, and since then, several functions have been proposed for it: adhesin, cell surface antigenic protein, and 3-carboxy-*cis*,*cis*-muconate cyclase. Experimental evidence has supported all these roles, but their relevance for the *Sporthrix*–host interaction remains elusive. The phenotypical characterization of the mutants generated here demonstrated the three functions that may be fulfilled by Gp70. The silenced strains showed a selective defect in the ability to bind fibronectin and laminin, which is in agreement with previous observations that suggested Gp70 binds to both extracellular matrix components [14,15]. It is noteworthy that Gp70 did not show a promiscuous binding to several extracellular matrix components, like Pap1 and Hsp60, two recently characterized adhesins belonging to *S. schenckii* peptidorhamnomannan [33]. These are two moonlighting proteins that are classified as intrinsically disordered proteins, a structural trait that may explain the broad ligand repertory for adherence [33]. The Gp70 bioinformatics analysis found that it is not considered an intrinsically disordered protein (analyzed at https://iupred2a.elte.hu; accessed on 18 March 2024), providing a possible explanation for the specific ability to bind only to laminin and fibronectin. It was previously demonstrated that both Pap1 and Hsp60 were responsible for about 50% of *S. schenckii*’s ability to bind laminin, whilst both contribute about 70% to fibronectin binding [33]. Here, the highly *GP70*-silenced strains showed a reduction of about 50% and 60% of binding to fibronectin and laminin, respectively. These data suggest that these three cell wall proteins are the main players in the binding of *S. schenckii* to both extracellular matrix components. Currently, it is not possible to elucidate whether Gp70 adhesive properties may depend on the interaction with other cell wall proteins, such as Pap1 and Hsp60. Nevertheless, our results indicate that Gp70 has adhesive properties that contribute to the *S. schenckii* interaction with the extracellular matrix.

3-carboxy-*cis*,*cis*-muconate cyclase activity has been related to the benzoate degradation pathway, which might be required during the saprophytic lifestyle when growing on decaying vegetable matter [18]. Here, we confirmed that Gp70 has this enzyme activity, and this was reduced to threshold levels in the highly *GP70*-silenced mutants. However, it remains to be addressed whether the enzyme has a fundamental role during the saprophytic stage. It is worth noting that the *GP70* expression analysis showed repression during filament growth [61], but this was stimulated in a rich and temperature-controlled medium, which might be different from the conditions imposed by the environment when growing on decaying matter.

In *S. schenckii*, it is thought that the cell wall integrity pathway is activated to respond against cell wall disturbances, such as those generated by genetic means or by incubating with toxic compounds, like Congo Red [26,48,62]. The increment in the β-1,3-glucan content and its exposure at the cell surface of the *GP70*-silenced strains, along with overexpression of the putative ortholog of *MCK1*, may be explained by the activation of this pathway. The fact that chitin content was not affected may indicate that the reduction in Gp70 levels is not drastic enough to activate generalized pathways that ended up in an increment of both kinds of structural polysaccharides. The changes in rhamnose, mannose, cell wall porosity, and binding to Alcian blue are in line with defects in the protein glycosylation pathway or changes in the levels of cell wall glycoproteins. It was previously established that Gp70 is an abundant cell wall protein that is distributed in pointed discrete patches all over the cell surface [20]. Thus, it is likely that the silenced mutants are displaying these phenotypes as a direct consequence of the decrement of Gp70 levels. 

The cytokine profile stimulated by the highly *GP70*-silenced mutant strains is similar to that observed in the *rmlD*-silenced mutant strain, where rhamnose levels are reduced and β-1,3-glucans levels incremented [26]. Again, similar to the *rmlD*-silenced mutants, here, TNFα and IL-6 production were dectin-1-, TLR2-, and TLR4-dependent, while IL-1β and IL-10 were exclusively dectin-1-dependent [26]. Thus, it is likely that the changes in cytokine production are related to the rhamnose reduction in the *GP70*-silenced strains. Similar to IL-1β and IL10 stimulation, phagocytosis by human granulocytes was exclusively dependent on dectin-1, an observation that fits with previous results reported for the interaction of neutrophils with *C. albicans* [63]. In the same sense, the increment in the ability to stimulate NETs is likely to be explained by the increment of β-1,3-glucan at the cell surface, as recently proposed by our group [64]. 

Despite our results showing virulence attenuation in *G. mellonella*, it is currently not clear whether this phenotype is a direct result of the reduction in the Gp70 levels, a reflection of the changes in the cell wall composition and, therefore, a different ability to interact with host components, including immune effectors, or the combination of these two aspects. It was previously proposed that the Gp70 levels at the cell wall negatively correlate with *Sporothrix* virulence [20]. Thus, a *GP70*-silenced strain is predicted to show a hypervirulent phenotype. However, it has also been proposed that strains with low rhamnose content showed low virulence compared with those with high rhamnose levels at the cell wall [56,65]. In light of these results, it is possible to speculate that a reduction in rhamnose levels rather than the cell wall Gp70 content explains the phenotype observed in the *GP70*-silenced mutants. Previous work on Gp70 localization at the cell wall was performed with yeast-like cells generated after 7 days of incubation at 37 °C [20], and it was previously demonstrated that cell wall composition and structures are different in *S. schenckii* yeast-like cells growing for 4 or 10 days in cultures [66]. Here, we used 4-day-old yeast-like cells, and this technical difference may account for the unpredicted results indicating virulence attenuation in the *GP70*-silenced mutants. Nevertheless, Gp70 is an important cell wall component whose absence affects the *Sporothrix*–host interaction.

In conclusion, we report here that the silencing of *S. schenckii GP70* led to defects in the cell adhesion to laminin and fibrinogen, changes in the cell wall composition, interaction with immune effectors, and virulence attenuation. Our results demonstrate that Gp70 is a versatile protein with adhesin properties, is responsible for the activity of 3-carboxy-*cis*,*cis*-muconate cyclase, and is a relevant protein during the *S. schenckii*–host interaction.

## Figures and Tables

**Table 1:** Analysis of *GP70* expression, binary plasmid insertional events, and 3-carboxy-*cis*,*cis*-muconate cyclase activity in control and silenced *Sporothrix schenckii* strains.

Strain	Relative *GP70* Expression (%) ^a^	GP70 Copy Number ^a^	Gp70 Enzyme Activity ^b^
Wild-type	100.0 ± 0.5	1.0 ± 0.1	21.2 × 10^−2^ ± 6.7 × 10^−2^
HSS39	98.2 ± 1.4	1.2 ± 0.3	19.8 × 10^−2^ ± 5.2 × 10^−2^
HSS40	99.4 ± 1.2	1.4 ± 0.3	18.0 × 10^−2^ ± 8.4 × 10^−2^
HSS41	46.7 ± 1.2 *	2.9 ± 0.3 *	7.2 × 10^−2^ ± 3.4 × 10^−2^ *
HSS42	38.6 ± 2.4 *	2.9 ± 0.4 *	6.4 × 10^−2^ ± 4.7 × 10^−2^ *
HSS43	37.2 ± 3.4 *	3.1 ± 0.3 *	7.7 × 10^−2^ ± 2.5 × 10^−2^ *
HSS44	1.8 ± 0.7 *	2.8 ± 0.2 *	No detected
HSS45	12.1 ± 1.6 *	3.1 ± 0.3 *	2.4 × 10^−2^ ± 1.6 × 10^−2^ *
HSS46	9.1 ± 0.7 *	3.1 ± 0.4 *	No detected

^a^ The L6 gene was used for data normalization. Data are means ± SD of three independent experiments performed in duplicate. ^b^ The 3-carboxy-*cis*,*cis*-muconate cyclase specific activity was defined as Δ_260nm_ min^−1^ mg protein^−1^. * *p* < 0.05 when compared to the wild-type, HSS39, or HSS40 strains.

**Table 2:** Fungal burden, cytotoxicity, hemocyte, melanin, and phenoloxidase levels in *Galleria mellonella* infected with *Sporothrix schenckii* wild-type, control, and *GP70*-silenced strains.

Strain	Colony-Forming Units (×10^5^) ^a^	Cytotoxicity (%) ^b^	Hemocytes (×10^6^) mL^−1^	Melanin ^c^	Phenoloxidase ^d^
PBS ^e^	0.0 ± 0.0	10.5 ± 3.3	3.6 ± 0.4	1.1 ± 0.5	0.4 ± 0.2
WT ^f^	3.2 ± 0.8	92.3 ± 7.7	7.9 ± 0.2	5.2 ± 0.4	3.6 ± 0.6
HSS39	3.1 ± 0.7	87.9 ± 10.5	7.8 ± 0.6	5.2 ± 0.6	3.3 ± 0.5
HSS40	3.4 ± 0.6	90.5 ± 8.4	8.2 ± 0.4	5.4 ± 0.3	3.7 ± 0.2
HSS41	2.9 ± 0.7	60.3 ± 8.9 *^,†^	5.4 ± 0.3 *^,†^	3.2 ± 0.5 *^,†^	2.4 ± 0.3 *^,†^
HSS42	3.1 ± 0.3	54.5 ± 10.4 *^,†^	5.3 ± 0.5 *^,†^	3.5 ± 0.7 *^,†^	2.1 ± 0.6 *^,†^
HSS43	3.3 ± 0.7	61.1 ± 9.9 *^,†^	5.0 ± 0.3 *^,†^	3.4 ± 0.5 *^,†^	2.3 ± 0.4 *^,†^
HSS44	3.2 ± 0.8	20.2 ± 9.5 *	3.9 ± 0.4 *	1.7 ± 0.2 *	1.0 ± 0.5 *
HSS45	3.4 ± 0.6	28.2 ± 8.6 *	4.0 ± 0.6 *	1.8 ± 0.6 *	0.8 ± 0.2 *
HSS46	3.1 ± 0.4	22.1 ± 7.5 *	3.8 ± 0.3 *	1.6 ± 0.7 *	1.2 ± 0.6 *

^a^ Animals were decapitated, and hemolymph was collected and used to calculate the colony-forming units by serial dilutions in YPD plates. ^b^ Lactate dehydrogenase activity quantified in cell-free hemolymph of infected larvae. The 100% activity corresponds to data obtained with lysed hemocytes. ^c^ Calculated in the cell-free hemolymph as A_405nm._ ^d^ Enzyme activity defined as the Δ_490nm_ min^−1^ μg protein ^−1^. ^e^ Larvae inoculated only with PBS. ^f^ WT, strain 1099-18 ATCC MYA 4821. * *p* < 0.05 when compared with the values obtained in animals infected with the WT, HSS39, or HSS40 strains. ^†^ *p* < 0.05 when compared with the measurements in animals infected with the WT or HSS44-HSS46 strains.

## Data Availability

Data are contained within the article.
